# Leaders development program by 360 degree feedback: reflection on head nurses’ leadership practices

**DOI:** 10.1186/s12912-024-02395-w

**Published:** 2024-10-21

**Authors:** Sabrine Mohammed Emam, Samah Faisal Fakhry, Hanaa Mohamed Abdrabou

**Affiliations:** Faculty of Nursing, Ain Shams University, Cairo, Egypt

**Keywords:** Leadership, 360-degree feedback, Nurse administrators, Professional development, Feedback, Psychological, Organizational culture

## Abstract

**Background:**

Leadership in nursing is crucial for delivering high-quality healthcare and ensuring positive outcomes for patients, staff, and institutions. Many nurses in leadership positions lack formal training, which can compromise their effectiveness. This study aims to evaluate the effect of a leadership development program utilizing 360-degree feedback on head nurses’ leadership practices.

**Methods:**

A true-experimental design was employed in three healthcare institutions. The study involved 80 head nurses (40 intervention, 40 control), 240 staff nurses, and 29 supervisors. The intervention group participated in a six-week leadership development program using 360-degree feedback. Data were collected pre- and post-intervention using the Leadership Development and 360-Degree Feedback Knowledge Questionnaire and the Leadership Practices Inventory (LPI).

**Results:**

The intervention group showed significant improvements in leadership knowledge and practices across all dimensions. Knowledge scores increased from 25.1 ± 8.8 to 93.0 ± 5.1 post-intervention, maintaining at 83.2 ± 7.1 at follow-up. Self-assessed leadership scores improved from 88.1 ± 6.0 to 97.5 ± 2.7, and 98.5 ± 2.0 at follow-up. Supervisor and staff assessments also showed substantial increases. Multiple linear regression analyses confirmed the strong positive impact of the intervention on leadership outcomes.

**Conclusion:**

The leadership development program using 360-degree feedback significantly enhanced head nurses’ leadership knowledge and practices. The results suggest that such programs can improve leadership capabilities in healthcare settings, leading to better patient care and organizational performance. Future research should address group homogeneity and explore long-term impacts on patient outcomes.

## Introduction

Leadership in nursing is crucial for delivering high-quality healthcare and ensuring positive outcomes for patients, staff, and institutions [1]. Despite its importance, many nurses in leadership positions often lack formal training, which can compromise their effectiveness and, consequently, the quality of care provided [2, 3]. Leadership development programs, therefore, have become a priority in the healthcare environment, aiming to equip nurse leaders with the necessary skills to manage and inspire their teams effectively [4].

One of the innovative approaches to leadership development is the use of 360-degree feedback, also known as multi-source feedback [5–7]. This method involves collecting comprehensive feedback on a leader’s performance from various sources, including peers, subordinates, and supervisors, providing a well-rounded view of their strengths and areas for improvement [8, 9].In previous studies, the 360-degree feedback approach has shown promise in enhancing self-awareness and improving leadership behaviors [10–12]. Despite this, there is a lack of consensus on the effectiveness of this method compared to other leadership development interventions. Moreover, existing literature often fails to address how the unique challenges of acute healthcare settings may influence the outcomes of such interventions [13, 14].

Despite the recognized importance of effective leadership in healthcare, the complexity and high-pressure environment of hospitals often pose significant challenges for nurse leaders [15]. Head nurses, in particular, must navigate diverse responsibilities, ranging from clinical oversight to administrative tasks and team management [16]. Traditional leadership training methods often overlook these multifaceted demands, focusing instead on generic management skills. This oversight can leave head nurses ill-prepared to handle the unique challenges of healthcare leadership, especially in specialized departments like critical care, where decision-making and team coordination are critical [17]. The incorporation of 360-degree feedback into leadership development programs offers a tailored approach that addresses these specific needs, providing actionable insights into a leader’s interpersonal and organizational skills [18].

This study’s findings will contribute to the existing body of literature on leadership development in healthcare by providing robust evidence on the impact of 360-degree feedback. The results are expected to inform future training programs, enhancing the leadership capacities of head nurses and ultimately improving patient care outcomes.

This study aims to fill this gap by assessing the effect of a leadership development program utilizing 360-degree feedback on the leadership practices of head nurses. Specifically, the study will investigate whether this approach leads to measurable improvements in leadership knowledge and practices. By focusing on head nurses across various hospital departments, including critical care units, this research will provide insights into the practical application and benefits of 360-degree feedback in a real-world healthcare environment.

## Aim of the study

The study aims to evaluate the effect of a leadership development program utilizing 360-degree feedback on head nurses’ leadership practices.

### Hypotheses

1. **Hypothesis 1**: *Participation in a leadership development program utilizing 360-degree feedback will significantly improve the leadership knowledge of head nurses compared to those who do not participate in the program.*
2. **Hypothesis 2**: *Head nurses who undergo the leadership development program will demonstrate significant improvements in their leadership practices*,* as measured by the Leadership Practices Inventory (LPI)*,* compared to head nurses who do not undergo the program.*
3. **Hypothesis 3**: *Head nurses who participate in the leadership development program will receive higher leadership performance ratings from their supervisors and staff nurses compared to those who do not participate in the program.*

### Methods

#### Design

A true-experimental design was employed in this study to evaluate the effect of a leadership development program using 360-degree feedback on the leadership practices of head nurses. The study utilized a randomized controlled trial (RCT) approach, where participants were randomly assigned to either the intervention group or the control group, ensuring comparability between the groups.

#### Setting

The study was conducted in three prominent healthcare institutions: Ain Shams University Hospital, a specialized pediatric hospital, and El-Demerdash Hospital. Ain Shams University Hospital, a major teaching hospital affiliated with one of Egypt’s oldest universities, offers a wide range of specialized medical services, making it ideal for studying leadership practices in high-stakes environments. The pediatric hospital focuses on the care of children and adolescents, providing a full spectrum of services from general care to specialized treatments. This setting requires unique leadership dynamics, particularly in communication and empathy, to support patient and family-centered care. El-Demerdash Hospital, known for its comprehensive medical and surgical services, includes critical care units that cater to patients needing advanced life support and continuous monitoring. The inclusion of these critical care settings allows for an examination of leadership practices in environments characterized by high patient acuity and multidisciplinary collaboration. The diversity in clinical departments and patient populations across these hospitals offers a robust platform for evaluating leadership practices among head nurses, ensuring that the study’s findings are applicable to a variety of healthcare contexts.

### Sample and sampling

The study employed a combination of proportional and disproportional stratified random sampling. For head nurses, a proportional sample ensured balanced representation, while for staff nurses, a disproportional approach was used to gather adequate feedback from a broader base of subordinates. The study involved a carefully structured sampling process to ensure representative data from key stakeholders in the healthcare setting. The sampling included head nurses, staff nurses, and supervisors, following a mixed proportional and disproportional stratified random sampling approach.

1. **Head Nurses**: A proportional sample of 80 head nurses was selected, with 40 head nurses assigned to the intervention group and 40 to the control group. This sample size was determined based on a moderate effect size (0.65) aiming for a 95% confidence level and 80% power in the study. The head nurses were selected to participate based on a minimum of two years of experience in their roles, ensuring a sufficient level of expertise and responsibility within the healthcare system.
2. **Staff Nurses**: A disproportional stratified random sample of 240 staff nurses was included, with 120 staff nurses allocated to each group. For each head nurse, three subordinate staff nurses were randomly selected to provide feedback and participate in the study. This selection method was also guided by the need to achieve a moderate effect size (0.40), maintaining a 95% confidence level and 80% study power. The involvement of staff nurses was crucial for evaluating the impact of the leadership development program on those directly supervised by the head nurses.
3. **Supervisors**: All available supervisors, totaling 29, were included in the study. These supervisors provided higher-level assessments and feedback as part of the 360-degree feedback process. The inclusion of all supervisors was essential to capture a comprehensive evaluation of the head nurses’ leadership practices from multiple perspectives.

### Inclusion and exclusion criteria

#### Inclusion criteria

- Participants (head nurses, staff nurses, and supervisors) with at least two years of experience in their respective roles.
- Consent to participate in the study, including attending all required sessions and completing assessments.

#### Exclusion criteria

- Individuals with prior participation in similar leadership development programs to avoid biases due to previous exposure.

##### Sample size calculation

**Fig. 1:**
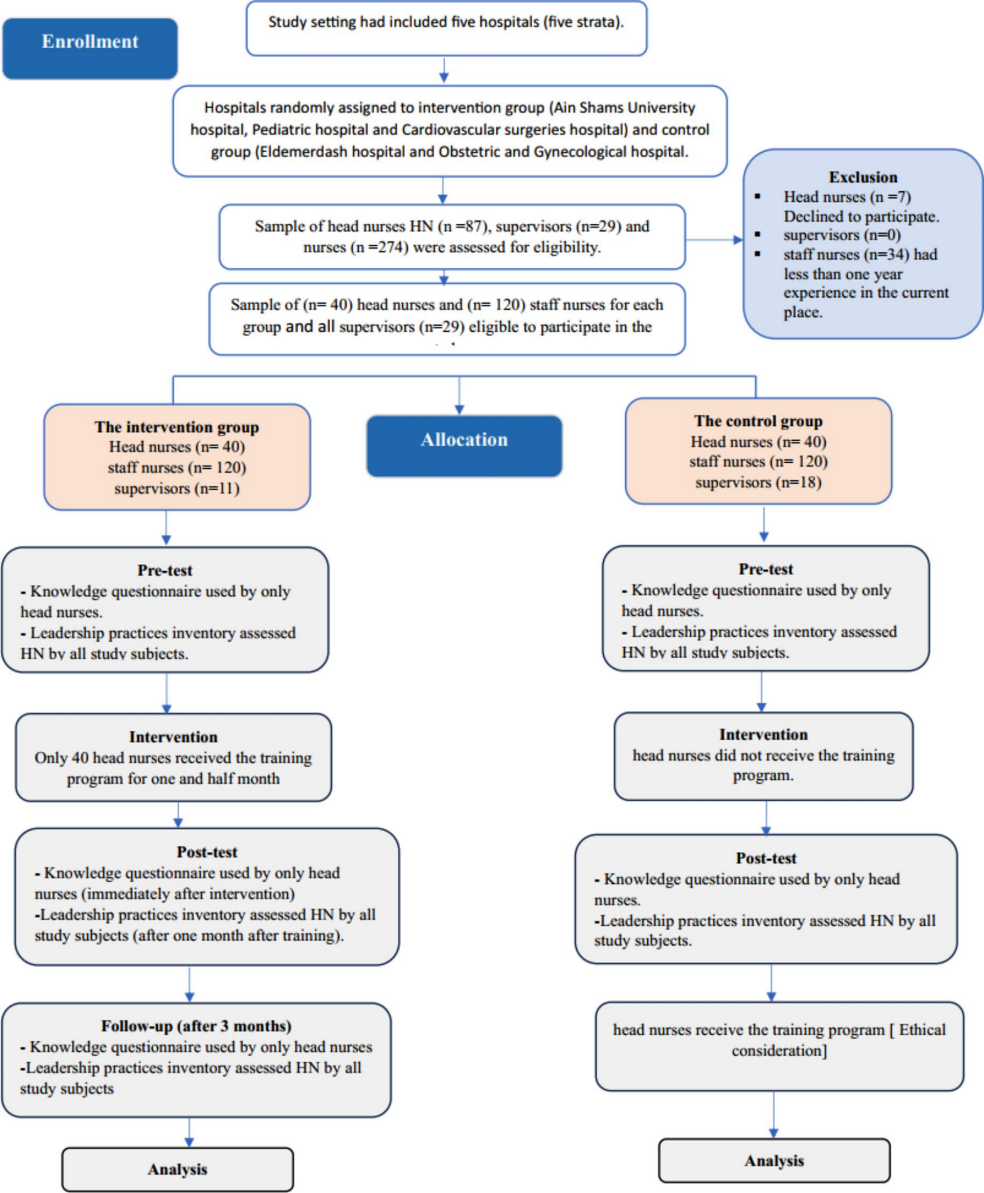
The sample flow chart

The sample size was determined using power analysis to ensure sufficient statistical power to detect meaningful differences between the intervention and control groups. Based on a desired power of 0.80, an alpha level of 0.05, and an expected moderate effect size (Cohen’s d = 0.50) for changes in leadership practices, the required sample size was calculated to be 80 head nurses. The same parameters were applied to calculate the sample size for assistant nurses and supervisors, resulting in a total of 240 assistant nurses and 40 supervisors. These calculations were grounded in prior studies and practical considerations within the similar healthcare setting [19–21]. The selection flow chat is shown in Fig. 1.

## Data collection tools

The data collection tools utilized in this study included the leadership development and 360-degree feedback assessment knowledge questionnaire, as well as the Leadership Practices Inventory (LPI).

### The Leadership Development and 360-Degree feedback knowledge questionnaire

The Leadership Development and 360-Degree Feedback Knowledge Questionnaire is a comprehensive tool designed to assess head nurses’ knowledge of leadership and 360-degree feedback mechanisms. originally developed by Beverly alimo-metcalfe (1998) [8] and adapted by the researcher, based on the works of Williams (2021) and Pavlik (2019) [22], and translated into Arabic by Hussein (2020) [23], this questionnaire features 42 multiple-choice questions across five dimensions: leadership and management concepts (8 questions), leadership styles (8 questions), leadership practices (13 questions), 360-degree feedback assessment (7 questions), and self-awareness (6 questions).

Each correct response earns one point, with scores for each dimension summed and converted into percentage scores. Knowledge is considered satisfactory if the score is 60% or higher. Validity was established through face and content validation by five nursing administration experts, leading to modifications for clarity and relevance. The tool demonstrated high reliability, with a Guttman split-half coefficient of 0.729.

This questionnaire serves as a robust and reliable instrument, providing valuable insights into the leadership capabilities of head nurses and facilitating targeted development and effective feedback mechanisms to enhance their leadership practices.

### The Leadership practices inventory (LPI)

The Leadership Practices Inventory (LPI) is a comprehensive tool developed by Kouzes and Posner in 2017 to assess leadership behaviors [24] and was translated into Arabic by Hussein in 2021 [23]. It comprises 30 self-administered statements categorized into five dimensions: “Model the Way,” “Inspire a Shared Vision,” “Challenge the Process,” “Enable Others to Act,” and “Encourage the Heart.” Each statement is rated on a 5-point Likert scale, ranging from “rarely” to “always.” For example, a statement might read, “I set a personal example of what I expect from others,” reflecting the “Model the Way” dimension. The scoring involves summing the scores for each dimension and calculating the mean to obtain percentage scores. Knowledge is considered satisfactory if the score is 60% or higher.

The LPI’s validity is well-established, supported by extensive research. It has demonstrated high internal consistency, with Cronbach’s alpha coefficients ranging from 0.73 to 0.95. In this study, the LPI showed a Cronbach’s alpha of 0.98, indicating extremely high reliability. While such a high value suggests that the items are highly correlated and may be measuring very similar constructs, it ensures a precise measure of specific leadership traits. Combining these variables into a single score provides a comprehensive and precise assessment of leadership practices, allowing for reliable inferences about the head nurses’ leadership capabilities. The tool provides valuable insights into leadership behaviors and areas for improvement, promoting self-awareness and effective leadership practices. It is widely used in both academic research and practical leadership development programs, helping leaders to enhance their organizational performance and team dynamics by offering a holistic view of their leadership capabilities. Through 360-degree feedback, the LPI facilitates targeted development and supports the continuous improvement of leadership skills.

## Operational definitions of variables

### Leadership Knowledge

- **Definition**: The understanding and awareness of concepts, styles, practices, and feedback mechanisms related to leadership among head nurses.
- **Measurement**: Assessed using the Leadership Development and 360-Degree Feedback Knowledge Questionnaire, which includes dimensions such as leadership and management concepts, leadership styles, leadership practices, 360-degree feedback assessment, and self-awareness. Each correct answer is scored as one point, with a total score converted into a percentage. Knowledge is considered satisfactory if the score is 60% or higher.

### Leadership practices

- **Definition**: The behaviors and actions exhibited by head nurses in their leadership roles, including modeling behavior, inspiring a shared vision, challenging processes, enabling others to act, and encouraging the heart.
- **Measurement**: Evaluated using the Leadership Practices Inventory (LPI), which consists of 30 self-administered statements rated on a 5-point Likert scale. Scores are summed and averaged to provide a percentage score for each dimension. The LPI’s validity and reliability are supported by high Cronbach’s alpha coefficients.

### 360-Degree feedback

- **Definition**: A comprehensive evaluation method that gathers feedback on an individual’s leadership performance from multiple sources, including supervisors, peers, and subordinates.
- **Measurement**: Incorporated into the leadership development program to provide a holistic assessment of head nurses’ leadership capabilities, identifying strengths and areas for improvement.

### Self-evaluation of performance

- **Definition**: The head nurses’ own assessment of their leadership practices and knowledge.
- **Measurement**: Part of the 360-degree feedback process and the Leadership Practices Inventory (LPI), where head nurses rate their own behaviors and practices.

### Field Work

The field work for this study involved several key phases, including preparation, implementation of the leadership development program, data collection, and follow-up. Each phase was meticulously planned and executed to ensure the integrity and reliability of the data collected.

### Preparation phase

Before the commencement of the intervention, a comprehensive orientation session was conducted for all participants, including head nurses, staff nurses, and supervisors. This session aimed to familiarize participants with the study’s objectives, procedures, and expectations. The orientation included detailed explanations of the 360-degree feedback mechanism, the roles of each participant, and the significance of their input. Informed consent was obtained from all participants, ensuring they understood their voluntary participation and the confidentiality of their responses.

Additionally, the research team coordinated with hospital administration and department heads to schedule the training sessions and data collection activities. This coordination ensured minimal disruption to the hospital’s daily operations and facilitated the smooth running of the study. Necessary materials, such as feedback forms, questionnaires, and training modules, were prepared and organized in advance.

### Implementation of the Leadership Development Program

The core component of the field work involved the implementation of a structured leadership development program for the intervention group. This program was designed based on the principles of the 360-degree feedback process and included multiple interactive training sessions. These sessions covered various aspects of leadership, including communication skills, conflict resolution, decision-making, and emotional intelligence. The training was delivered through a combination of lectures, workshops, and role-playing exercises, aiming to enhance the head nurses’ leadership competencies.

Each training session was facilitated by experienced trainers who specialized in healthcare leadership. The sessions were conducted over a period of six weeks, with weekly meetings lasting three hours each. The content was tailored to address the specific challenges faced by head nurses in a hospital setting. During these sessions, participants were encouraged to engage actively, share their experiences, and discuss practical applications of the leadership concepts being taught.

### Data Collection

Data collection was conducted in two main phases: pre-intervention and post-intervention.

#### Pre-intervention Data Collection

Prior to the start of the training program, baseline data were collected from all participants. Head nurses, staff nurses, and supervisors completed the Leadership Practices Inventory (LPI) and the Leadership Development and 360-Degree Feedback Knowledge Questionnaire. These instruments were used to assess the current level of leadership knowledge and practices among head nurses, as well as perceptions of their leadership effectiveness from the perspective of staff nurses and supervisors.

#### Post-intervention Data Collection

Following the completion of the leadership development program, the same questionnaires were administered to all participants to measure any changes in leadership knowledge and practices. The post-intervention data collection took place one month after the final training session, providing sufficient time for the participants to apply and reflect on the skills and knowledge gained.

### Follow-Up and Feedback

Following the completion of the leadership development program and the post-intervention data collection, a follow-up phase was conducted three months later. This follow-up aimed to assess the long-term impact of the program on head nurses’ leadership practices. The same questionnaires used in the pre- and post-intervention phases were administered to the participants to evaluate the retention and application of the skills and knowledge gained during the program. This phase was critical in determining the sustainability of the program’s effects and providing insights into areas requiring ongoing support and reinforcement.

### Data Analysis and Reporting

The collected data were systematically analyzed to identify significant differences between the pre- and post-intervention scores, as well as between the intervention and control groups. This analysis helped in evaluating the effectiveness of the leadership development program. The findings were compiled into a detailed report, highlighting key outcomes, insights, and recommendations for future leadership training initiatives within the healthcare sector.

The field work was crucial in gathering comprehensive and actionable data on the impact of the leadership development program. It provided valuable insights into the enhancement of leadership skills among head nurses and the overall improvement in the quality of care within the hospital setting. The structured and well-coordinated field work ensured the successful completion of the study and contributed significantly to the understanding of effective leadership practices in healthcare.

### Statistical analysis

Data entry and statistical analysis were performed using the SPSS 20.0 statistical software package. Descriptive statistics, such as frequencies and percentages for qualitative variables, and means, standard deviations, and medians for quantitative variables, were used to present the data. The reliability of the knowledge and leadership tools was assessed using Split-half Gutman and Cronbach’s alpha coefficients, respectively. F-tests were utilized to compare quantitative continuous data, while chi-square tests were used for qualitative categorical variables. Spearman rank correlation was employed to assess the interrelationships among quantitative and ranked variables. Multiple linear regression analysis was conducted to identify independent predictors of knowledge and leadership scores, with analysis of variance for the full regression models. Statistical significance was determined at a p value < 0.05.

## Results

Table 1 presents the demographic characteristics of head nurses in the study and control groups, revealing several notable differences. The study group had a significantly higher average age (48.9 ± 4.9) compared to the control group (44.7 ± 6.8), with a p-value of 0.002, based on the t-test comparing mean values. There was also a significant difference in marital status distribution, with a higher proportion of married individuals in the control group (97.5%) compared to the study group (82.5%), as indicated by a p-value of 0.03 from the chi-square test. Additionally, the study group consisted exclusively of female participants, similar to the control group, with no significant difference in gender distribution (*p* = 0.31). Educational qualifications were comparable between the groups, predominantly consisting of diploma holders. A significant disparity was observed in hospital affiliation, with the study group representing various hospitals (Ain-Shams University, Pediatrics, El-Demerdash) and the control group exclusively from CV surgery and Ob/Gyne units (*p* < 0.001). Critical unit experience was more prevalent among the study group (67.5%) compared to the control group (40.0%), with a significant p-value of 0.01, also based on the chi-square test. The groups were similar in terms of years of experience as head nurses and overall experience. However, the study group had a higher proportion of head nurses with 30 + years of total experience (52.5% vs. 20.0%, *p* = 0.002), with this comparison again based on mean values. Prior leadership training was more common in the study group, but this difference was not statistically significant (*p* = 0.14).

**Table 1:** Demographic characteristics of head nurses in the study and control groups

	Group				X^2^ test	*p*-value
	Study (*n* = 40)		Control (*n* = 40)			
	No.	%	No.	%		
Age:						
< 50	21	52.5	28	70.0		
50+	19	47.5	12	30.0	2.58	0.11
Range	40–59		32–59			
Mean ± SD	48.9 ± 4.9		44.7 ± 6.8		t = 3.17	0.002*
Median	49.0		43.5			
Gender:						
Male	1	2.5	0	0.0		
Female	39	97.5	40	100.0	1.01	0.31
Marital status:						
Unmarried	7	17.5	1	2.5		
Married	33	82.5	39	97.5	5.00	0.03*
Nursing qualification:						
Diploma	31	77.5	33	82.5		
Bachelor	9	22.5	7	17.5	0.31	0.58
Hospital:						
Ain-Shams University	15	37.5	0	0.0		
Pediatrics	15	37.5	0	0.0		
El-Demerdash	10	25.0	0	0.0	80.00	< 0.001*
CV surgery	0	0.0	25	62.5		
Ob/Gyne	0	0.0	15	37.5		
Unit:						
Critical	27	67.5	16	40.0		
Non-critical	13	32.5	24	60.0	6.08	0.01*
Experience years (current as HN):						
< 10	16	40.0	18	45.0		
10+	24	60.0	22	55.0	0.21	0.65
Range	< 1 = 35		1–39			
Mean ± SD	14.0 ± 10.2		13.9 ± 9.4		t = 0.07	0.95
Median	12.0		11.5			
Experience years (total):						
< 30	19	47.5	32	80.0		
30+	21	52.5	8	20.0	9.14	0.002*
Range	20–40		8–40			
Mean ± SD	29.0 ± 4.9		24.0 ± 6.9		3.74	< 0.001*
Median	30.0		23.5			
Had training in leadership	15	37.5	9	22.5	2.14	0.14

Table 2 illustrates the scores of knowledge and leadership perception among head nurses in the study group throughout the intervention. Significant improvements were noted in all domains post-intervention, with knowledge scores increasing from 25.1 ± 8.8 to 93.0 ± 5.1 and slightly decreasing to 83.2 ± 7.1 at follow-up, but remaining significantly higher than pre-intervention scores (*p* < 0.001). Similarly, self-assessed leadership, supervisor-assessed leadership, and staff-assessed leadership scores showed substantial increases post-intervention, maintaining significant gains at follow-up (*p* < 0.001), indicating the program’s effectiveness.

**Table 2:** Scores of knowledge and leadership perception in the study group throughout the intervention

	TIME			F-Test	*p*-value
	Pre	Pot	FU		
	Mean ± SD	Mean ± SD	Mean ± SD		
Knowledge	25.1 ± 8.8	93.0 ± 5.1	83.2 ± 7.1	1050.78	< 0.001*
Leadership (self)	88.1 ± 6.0	97.5 ± 2.7	98.5 ± 2.0	83.82	< 0.001*
Leadership (supervisor)	61.5 ± 14.0	91.0 ± 6.7	89.3 ± 5.9	119.52	< 0.001*
Leadership (staff)	44.3 ± 8.7	86.7 ± 5.5	86.1 ± 6.7	496.55	< 0.001*

(*) Statistically significant at *p* < 0.05

Table 3 shows that the control group’s scores for knowledge and leadership perception remained unchanged throughout the intervention. The mean scores for knowledge (24.9 ± 8.6 pre, 24.9 ± 8.5 post), self-assessed leadership (89.9 ± 5.80 pre, 90.0 ± 5.8 post), leadership as assessed by supervisors (68.5 ± 11.8 pre, 68.5 ± 11.8 post), and leadership as assessed by staff (53.6 ± 14.3 pre, 53.6 ± 14.3 post) showed no significant differences, with p-values indicating no statistical significance (*p* > 0.98).

**Table 3:** Scores of knowledge and leadership perception in the control group throughout the intervention

	TIME		F-Test	*p*-value
	Pre	Post		
	Mean ± SD	Mean ± SD		
Knowledge	24.9 ± 8.6	24.9 ± 8.5	0.00	0.98
Leadership (self)	89.9 ± 5.80	90.0 ± 5.8	0.00	0.98
Leadership (supervisor)	68.5 ± 11.8	68.5 ± 11.8	0.00	1.00
Leadership (staff)	53.6 ± 14.3	53.6 ± 14.3	0.00	1.00

The multiple linear regression model for head nurses’ knowledge scores, as shown in Table 4, indicates significant predictors. The intervention group had a positive standardized coefficient (B = 23.23, *p* < 0.001), showing a strong positive impact on knowledge scores. Conversely, the control group had a negative coefficient (B = -30.56, *p* < 0.001), reflecting lower knowledge scores. The model explains 68% of the variance (r-square = 0.68). The model’s overall fit was highly significant (F = 209.07, *p* < 0.001), with variables such as age, gender, qualification, marital status, experience, hospital, unit, and courses considered.

**Table 4:** Best fitting multiple linear regression model for head nurses’ knowledge score

	Unstandardized Coefficients		Standardized Coefficients	t-test	*p*-value	95% Confidence Interval for B	
	B	Std. Error				Lower	Upper
Constant	51.19	6.01		8.521	< 0.001	39.35	63.04
Control group	-30.56	2.79	-0.47	-10.960	< 0.001	-36.06	-25.06
Intervention	23.23	1.83	0.54	12.724	< 0.001	19.63	26.83

r-square = 0.68, Model ANOVA: F = 209.07, *p* < 0.001

Variables entered and excluded: age, gender, qualification, marital status, experience, hospital, unit, courses

Table 5 presents the best-fitting multiple linear regression model for the leadership (self) score, showing significant predictors. The constant is 62.33 (*p* < 0.001). The intervention has a coefficient of 2.17 (*p* = 0.001), female gender 7.26 (*p* = 0.009), total experience 0.24 (*p* < 0.001), and knowledge score 0.08 (*p* < 0.001). The model’s r-square is 0.48, indicating it explains 48% of the variance in self-assessed leadership scores. The model ANOVA is significant (F = 35.32, *p* < 0.001), validating the predictors’ collective contribution.

**Table 5:** Best fitting multiple linear regression model for the leadership (self) score

	Unstandardized Coefficients		Standardized Coefficients	t-test	*p*-value	95% Confidence Interval for B	
	B	Std. Error				Lower	Upper
Constant	62.33	5.90		10.558	< 0.001	50.69	73.98
Intervention	2.17	0.62	0.25	3.498	0.001	0.95	3.40
Female gender	7.26	2.77	0.14	2.622	0.009	1.80	12.71
Total experience	0.24	0.06	0.23	4.275	< 0.001	0.13	0.35
Knowledge score	0.08	0.02	0.43	5.412	< 0.001	0.05	0.12

r-square = 0.48, Model ANOVA: F = 35.32, *p* < 0.001

Variables entered and excluded: age, qualification, marital status, current experience, unit, courses, hospital

The multiple linear regression model for the leadership (supervisor) score revealed several significant predictors (Table 6). The intervention had a positive effect (B = 3.67, *p* = 0.007). Age was a negative predictor (B = -0.28, *p* = 0.038), while being married (B = 5.18, *p* = 0.036) and holding a bachelor’s degree (B = 6.17, *p* = 0.001) were positive predictors. Course attendance negatively impacted the score (B = -4.45, *p* = 0.009), and the knowledge score was a strong positive predictor (B = 0.31, *p* < 0.001). The model explained 62% of the variance (r-square = 0.62).

**Table 6:** Best fitting multiple linear regression model for the leadership (supervisor) score

	Unstandardized Coefficients		Standardized Coefficients	t-test	*p*-value	95% Confidence Interval for B	
	B	Std. Error				Lower	Upper
Constant	58.04	7.82		7.418	< 0.001	42.61	73.47
Intervention	3.67	1.35	0.17	2.725	0.007	1.01	6.32
Age	-0.28	0.13	-0.11	-2.089	0.038	-0.54	-0.02
Married	5.18	2.45	0.10	2.114	0.036	0.35	10.00
Bachelor degree	6.17	1.83	0.16	3.378	0.001	2.57	9.78
Course attendance	-4.45	1.68	-0.13	-2.645	0.009	-7.76	-1.13
Knowledge score	0.31	0.03	0.63	9.161	< 0.001	0.24	0.38

r-square = 0.62 Model ANOVA: F = 37.70, *p* < 0.001

Variables entered and excluded: age, gender, qualification, marital status, experience, hospital

The results presented in Table 7 show the best-fitting multiple linear regression model for the leadership (staff) score. The constant term has a coefficient of 90.79 (SE = 14.98), which is highly significant (t = 6.060, *p* < 0.001). The intervention variable has a positive coefficient of 12.29 (SE = 2.06), indicating a significant positive impact on leadership scores (t = 5.960, *p* < 0.001). Age has a negative coefficient of -0.78 (SE = 0.31), suggesting a significant negative association (t = -2.508, *p* = 0.015). The model explains 39% of the variance in leadership scores (r-square = 0.39), and the overall model is significant (F = 20.69, *p* < 0.001).

**Table 7:** Best fitting multiple linear regression model for the leadership (staff) score

	Unstandardized Coefficients		Standardized Coefficients	t-test	*p*-value	95% Confidence Interval for B	
	B	Std. Error				Lower	Upper
Constant	90.79	14.98		6.060	0.000	60.88	120.70
Intervention	12.29	2.06	0.58	5.960	0.000	8.17	16.41
Age	-0.78	0.31	-0.24	-2.508	0.015	-1.41	-0.16

r-square = 0.39 Model ANOVA: F = 20.69, *p* < 0.001

Variables entered and excluded: gender, qualification, marital status, experience, hospital, unit, group

## Discussion

This study aimed to evaluate the effect of a leadership development program utilizing 360-degree feedback on head nurses’ leadership practices. The findings demonstrate that the program had a significant positive impact on head nurses’ leadership knowledge and practices across all measured dimensions. These results highlight the potential of structured leadership training incorporating multi-source feedback to enhance nursing leadership capabilities in healthcare settings.

### Impact on Leadership Knowledge

The significant improvements observed in the intervention group’s leadership knowledge scores, rising from 25.1 ± 8.8 pre-intervention to 93.0 ± 5.1 post-intervention and maintaining at 83.2 ± 7.1 at follow-up, underscore the program’s effectiveness in increasing participants’ understanding of leadership concepts and practices. This aligns with previous researches found that formal leadership development programs positively influence individual competencies among hospital leaders [25, 26]. The sustained improvement at follow-up suggests that the knowledge gained was not merely short-term, but had a lasting impact on participants’ leadership understanding [27, 28].

The multiple linear regression analyses provide valuable insights into the factors influencing leadership outcomes. The strong positive impact of the intervention on knowledge scores (B = 23.23, *p* < 0.001) in the regression model confirms the program’s effectiveness in enhancing leadership knowledge. The negative coefficient for the control group (B = -30.56, *p* < 0.001) further emphasizes the contrast between those who received the intervention and those who did not.

### Improvement in Leadership practices

The substantial enhancements in self-assessed leadership scores, from 88.1 ± 6.0 pre-intervention to 97.5 ± 2.7 post-intervention and 98.5 ± 2.0 at follow-up, indicate increased confidence and self-awareness among head nurses regarding their leadership abilities. This growth in self-perception is a crucial outcome, as leaders’ self-awareness is closely linked to their effectiveness [29]. The consistency between self-assessments and evaluations from supervisors and staff further validates these improvements, suggesting that the changes were not only perceived by the head nurses themselves but were also evident to those they work with and lead [30–32]. Additionally, these findings resonate with Abdelaliem et al. (2024), who highlighted the importance of leader expertise and personal characteristics in influencing leadership effectiveness and reducing turnover intentions among nurses [33].

The regression model for supervisor-assessed leadership scores revealed several interesting predictors. The positive effect of the intervention (B = 3.67, *p* = 0.007) was accompanied by positive associations with being married and holding a bachelor’s degree. The negative impact of age suggests that younger head nurses may have been more receptive to the training or that supervisors may have higher expectations for more experienced nurses [34]. The negative association with prior course attendance is intriguing and may indicate that the 360-degree feedback approach was particularly beneficial for those without extensive prior leadership training [35].

For self-assessed leadership scores, the intervention emerged as a significant positive predictor (B = 2.17, *p* = 0.001), along with female gender, total experience, and knowledge scores. This suggests that while the program was effective across participants, individual characteristics and prior experience also play a role in leadership development outcomes [36, 37]. The positive association between knowledge scores and self-assessed leadership (B = 0.08, *p* < 0.001) indicates that increased understanding of leadership concepts translates to greater confidence in one’s leadership abilities [38].

### Enhanced Leadership performance ratings

The marked improvement in leadership assessments by supervisors (from 61.5 ± 14.0 to 91.0 ± 6.7) and staff nurses (from 44.3 ± 8.7 to 86.7 ± 5.5) provides strong evidence of the program’s impact on observable leadership behaviors. These findings are particularly noteworthy as they represent evaluations from diverse perspectives within the healthcare hierarchy, offering a comprehensive view of leadership improvement. The alignment of these assessments with the head nurses’ self-evaluations suggests that the program fostered not only improved leadership skills but also enhanced self-awareness and accurate self-perception among participants [39, 40].

The regression model for supervisor-assessed leadership scores revealed several interesting predictors. The positive effect of the intervention (B = 3.67, *p* = 0.007) was accompanied by positive associations with being married and holding a bachelor’s degree. The negative impact of age suggests that younger head nurses may have been more receptive to the training or that supervisors may have higher expectations for more experienced nurses [34]. The negative association with prior course attendance is intriguing and may indicate that the 360-degree feedback approach was particularly beneficial for those without extensive prior leadership training [35].

For staff-assessed leadership scores, the intervention again emerged as a strong positive predictor (B = 12.29, *p* < 0.001), with age showing a negative association. This consistency across different assessment perspectives reinforces the program’s effectiveness while highlighting the complex interplay of factors influencing leadership perceptions [41].

### Broader implications and alignment with existing literature

The findings align with previous research on the benefits of 360-degree feedback in leadership development. For instance, Sureda (2021) found that multi-source feedback enhanced motivation and performance among healthcare professionals in Iceland [42]. Similarly, Chandler et al. (2010) demonstrated the usefulness of 360-degree evaluations in outpatient settings, highlighting its value across various healthcare contexts [43].

The study’s results also support the broader literature on the importance of leadership development in nursing. As Kwame et al. (2024) emphasized, effective nursing leadership is crucial for delivering high-quality healthcare and ensuring positive outcomes for patients, staff, and institutions [44]. The significant improvements observed across multiple leadership dimensions in this study suggest that structured programs like the one implemented can address the leadership training gap often found in nursing [45] .

The holistic nature of the 360-degree feedback approach used in this study aligns with recommendations from Dalvi et al. (2023), who emphasized the role of comprehensive feedback as a management tool in performance improvement [46]. By incorporating perspectives from peers, subordinates, and supervisors, the program provided head nurses with a well-rounded view of their leadership strengths and areas for improvement, addressing the multifaceted demands of their roles [47]. A recent study by Spears-Jones et al. (2021) further supports the value of integrating 360-degree feedback in leadership training programs, emphasizing its effectiveness in enhancing leadership behaviors and performance outcomes across various organizational settings [48].

### Implications for nursing practice

The findings of this study have significant implications for nursing practice, particularly in leadership development. The positive impact of the 360-degree feedback-based leadership development program on head nurses’ leadership knowledge and practices suggests that such programs can play a crucial role in enhancing leadership capabilities in healthcare settings. Improved leadership practices among head nurses are likely to lead to better team dynamics, fostering a collaborative work environment essential for high-quality patient care. By incorporating 360-degree feedback into leadership training, healthcare institutions can cultivate a culture of continuous feedback and leadership development, which can significantly enhance decision-making, conflict resolution, and team management skills among nurse leaders. Moreover, effective leadership, as developed through these programs, is directly linked to improved patient outcomes. By enhancing the leadership competencies of head nurses, healthcare organizations can ensure that their teams are better equipped to meet the challenges of patient care, ultimately leading to better patient outcomes. The study also underscores the importance of sustainable professional growth. Integrating 360-degree feedback into regular professional development encourages ongoing personal and professional growth, ensuring that nurse leaders maintain and enhance their skills over time.

Furthermore, these findings suggest a need for healthcare institutions to invest in structured leadership development programs as part of their policy framework. Institutionalizing such programs can ensure that leadership development is a continuous process rather than a one-time event, leading to sustained improvements in leadership quality across the organization. Overall, the implementation of 360-degree feedback in leadership development programs is a valuable strategy that can lead to enhanced leadership practices, improved team performance, and better patient care outcomes.

## Limitations of the study

This study has several limitations that should be acknowledged. First, although efforts were made to ensure group comparability through randomization, the initial differences in age and experience between the intervention and control groups might have influenced the outcomes. These demographic differences could potentially affect the generalizability of the findings. Second, the reliance on self-reported data, particularly in the self-assessment of leadership practices, may introduce bias. Participants might have over- or under-estimated their leadership abilities, which could affect the accuracy of the results. While the study included multiple sources of feedback to mitigate this issue, self-perception remains a subjective measure.

Third, the study was conducted in a specific healthcare context within three institutions in Egypt, which may limit the applicability of the results to other settings or regions. Different cultural, organizational, and healthcare environments might influence how leadership development programs are perceived and their effectiveness. Fourth, the study did not fully account for the varying demands of different work environments, such as acute versus non-acute care settings. This variability might have affected the results, as leadership challenges and requirements can differ significantly between these environments.

Lastly, the follow-up period was relatively short, limited to three months after the intervention. While this period was sufficient to assess immediate and short-term impacts, it may not capture the long-term sustainability of the leadership improvements observed. Future research should include longer follow-up periods to evaluate the enduring effects of such programs.

## Conclusion of the study

This study provides robust evidence that a leadership development program using 360-degree feedback can effectively improve the leadership knowledge and practices of head nurses. The significant improvements observed in the intervention group, sustained through the follow-up phase, highlight the value of such programs in healthcare settings. Despite the study’s limitations, the findings support the integration of structured leadership development initiatives to enhance leadership capabilities, ultimately leading to better patient care and organizational performance.

The positive impact on leadership practices as perceived by both supervisors and staff nurses underscores the importance of comprehensive feedback mechanisms. These programs not only enhance self-awareness among head nurses but also promote a culture of continuous professional development. To maximize the benefits, healthcare organizations should consider implementing regular leadership training programs and addressing the specific needs of different groups within their workforce.

Future research should focus on addressing the limitations identified in this study, such as ensuring more homogeneous groups and using objective performance measures. Additionally, exploring the long-term impact of leadership development programs on patient care outcomes and organizational performance would provide valuable insights into their effectiveness and inform the design of future interventions.

## Data Availability

all required data are included in the research.

## Abbreviations

LPIs: Leadership Practices Inventory
HN: Head Nurse
SOA: Self-other Agreement
